# Epstein-Barr Virus EBER Transcripts Affect miRNA-Mediated Regulation of Specific Targets and Are Processed to Small RNA Species

**DOI:** 10.3390/ncrna1030170

**Published:** 2015-09-30

**Authors:** Julia Alles, Daniele Hasler, Syed Mohammad Ali Kazmi, Mathias Tesson, Andrew Hamilton, Linda Schlegel, Stefanie Marx, Norbert Eichner, Richard Reinhardt, Gunter Meister, Joanna B. Wilson, Friedrich A. Grässer

**Affiliations:** 1 Institute of Virology, Saarland University Medical School, Kirrbergerstrasse, Haus 47, Homburg/Saar D-66421, Germany; E-Mails: ju.al@mx.uni-saarland.de (J.A.); lindaschlegel@gmx.de (L.S.); stefanie.marx@uks.eu (S.M.); 2 Biochemistry Center Regensburg (BZR), Laboratory for RNA Biology, University of Regensburg, Universitätsstrasse 31, Regensburg D-93053, Germany; E-Mails: Daniele.Hasler@vkl.uni-regensburg.de (D.H.); Norbert.Eichner@vkl.uni-regensburg.de (N.E.); Gunter.Meister@vkl.uni-regensburg.de (G.M.); 3 College of Medical, Veterinary and Life Sciences, University of Glasgow, Glasgow G12 8QQ, UK; E-Mails: alikazmi39@gmail.com (S.M.A.K.); Mathias.Tesson@glasgow.ac.uk (M.T.); Andrew.Hamilton@glasgow.ac.uk (A.H.); 4 Max Planck Genome Centre Cologne, Carl-von-Linné-Weg 10, Cologne D-50829, Germany; E-Mail: reinhardt@mpipz.mpg.de (R.R.)

**Keywords:** Epstein-Barr virus, EBV, EBER, microRNA, DICER, Lupus antigen, La, ebv-miR-BART16, hsa-miR-142, IL1A, RAC1, TOMM22, ADCY9

## Abstract

The oncogenic Epstein-Barr virus (EBV) expresses 44 mature microRNAs and two non-coding EBER RNAs of 167 (EBER1) and 172 (EBER2) nt length. MiRNA profiling of NK/T cell lines and primary cells and Northern blotting of EBV-infected cell lines and primary tumors revealed processing of EBER1 to short 5′-derived RNAs of approximately 23, 52 and 70 nt (EBER1_23_, EBER1_52_, and EBER1_70_) and of EBER2 to 3′ fragments. The biogenesis of these species is independent of Dicer, and EBER1_23_ does not act like a miRNA to target its complementary sequence. EBER1, EBER2 and EBER1_23_ were bound by the lupus antigen (La), a nuclear and cytoplasmic protein that facilitates RNAi. Consistent with this, the EBERs affect regulation of interleukin 1alpha (IL1α) and RAC1 reporters harboring miR target sequences, targets of miR-142-3p. However, the EBERs have no effect upon another target of miR-142-3p, ADCY9, nor on TOMM22, a target of ebv-miR-BART16, indicative of selective modulation of gene expression by the EBERs.

## 1. Introduction

The Epstein-Barr virus (EBV) infects approximately 95% of the adult human population worldwide and generally establishes a symptomless, latent infection [1]. Under certain conditions, however, it induces malignant tumors of B- or T-cells, including Burkitt’s lymphoma (BL), Hodgkin’s lymphoma (HL) and nasal NK/T-cell lymphoma (NKTL), as well as epithelial tumors such as nasopharyngeal carcinoma (NPC) and gastric carcinoma (GC) [2]. The tumorigenic potential is reflected in its ability to readily transform B-cells into permanently growing cell lines (lymphoblastoid cell lines; LCLs), the *in vitro* equivalent of post-transplant lymphoproliferative disease (PTLD) that can arise in immunosuppressed patients [3]. In addition to protein-encoding genes, EBV was the first virus where microRNAs (miRNAs) were described [4] and these play important roles in transformation by EBV [5,6,7]. MiRNAs are conserved, small non-coding RNAs of approximately 22 nt length. They repress gene expression through binding to partially complementary sequences usually located in the 3’ untranslated region (UTR) of target mRNAs [8]. To carry out their regulatory functions, miRNAs are incorporated into RNA-induced silencing protein complexes (RISC) where they directly interact with a member of the Argonaute (Ago) protein family [9]. Upon binding to their distinct target sites, miRNA-Argonaute complexes recruit a member of the glycine-tryptophan-rich motif (GW) protein family, which recruits the deadenylation machinery leading to poly(A) tail shortening and finally mRNA decay. At early stages of repression, however, the GW protein coordinates translational repression of the mRNA without considerably affecting mRNA stability [10,11,12]. Like cellular miRNAs, viral miRNAs can be secreted in endosome-derived exosomes, and these show an enrichment for 3’end uridylated isoforms [13]. RISC complexes are associated with endosomal membranes [14,15] and knock down of GW182 reduces miRNA exosomal secretion [16], linking the mechanisms of miRNA activity and release.

In addition to miRNAs, EBV encodes two non-polyadenylated RNAs (Epstein-Barr virus Encoded RNA; EBER), spaced 160 bp apart in the viral genome [17]. These are transcribed by RNA polymerase III generating EBER1 and EBER2 of 167 and 172 nucleotides (nt), respectively, and terminate with four uracil nucleotides. The EBER transcripts are detectable at up to 10^7^ copies/cell in virtually all EBV-transformed tumors and cell lines [17,18]. Their high degree of conservation between viral strains suggests they perform an important function [19]. The EBER transcripts are detectable predominantly in the nucleus [20] although their presence in the cytoplasm has been reported [21] and they have been hypothesized to be secreted in exosomes through association with lupus antigen (Sjogren syndrome antigen B, or La) [22]. It is believed that they form double-stranded (ds) RNA structures with several hairpin loops [23]. Intriguingly, the EBERs can functionally substitute for the adenovirus VA transcripts during adenovirus replication; the VA transcripts also form dsRNA-structures [24] suggesting a broader role of these RNAs in viral infection. In addition to La [17], the EBERs associate with several cellular proteins, including double-stranded RNA-activated protein kinase R (PKR) [25], ribosomal protein L22 [26], hnRNP-D/AUF1 [27] and retinoic acid inducible gene I (RIG-I) [28]. Binding of the EBERs activates RIG-I and subsequently triggers the production of type I interferon and the activation of NFκB [29]. The interferon response can be induced by each EBER RNA separately with EBER1 showing a stronger activity. Although the EBERs are not essential for *in vitro* transformation of B-cells [30], EBER-deleted EBV has a significantly lower transformation potential as fewer cell clones grow out and these grow slower than wt-transformed B-cells [31]. Several reports suggest the EBERs have an important role in tumorigenic processes *in vivo* (reviewed in [32]). For example, the EBERs supply an autocrine growth signal via the secretion of insulin-like growth factor (IGF-1) from EBV-positive gastric carcinoma [33] and nasopharyngeal carcinoma [34], probably by conferring an anti-apoptotic signal to the cells [35]. Additionally, the release of EBERs complexed with La from EBV-infected cells can activate signaling from the toll-like receptor 3 (TLR3) [17,22]. Furthermore, transgenic mice expressing EBER1 develop lymphoid hyperplasia and show an increased lymphoma incidence, emphasizing the contribution of EBER1 to tumorigenesis [36]. Most recently, it was shown that the expression of the EBERs leads to deregulation of cellular protein expression [37].

Here, we show that the EBERs influence the target regulation of several miRNAs. Also, the EBERs not only exist in their full length forms, but also as shorter fragments of 70, 52 and 20–23 nt in size. Both the full length and the EBER1 20–23 nt fragment are bound by La. Since La is a classical RNA-binding protein (RBP), we hypothesize that EBER:La might affect miRNA-mediated regulation within a specific mRNP context.

## 2. Material and Methods

### 2.1. Cell Culture

All cell lines (including HaCaT) were cultured as described previously [38,39,40,41,42,43]. HEK293 cells that contain an EBER-deleted EBV [44], an EBV-transformed lymphoblastoid cell line AM29 (parental EBV-strain with EBERs) and derivative AM58 (EBERs deleted) [30] were used in these studies. Dicer-deficient mouse embryonic fibroblasts (MEF) cells [41] were transfected in a 24-well format with 500 ng of plasmid using Lipofectamine LTX reagent (Life technologies, Grand Island, NY, USA). HeLa cells were transfected using Fugene 6 (Roche, Mannheim, Germany).

### 2.2. RNA Sequencing

RNA-Seq libraries were prepared according to supplier recommendations (TruSeq DNA/RNA sample preparation v2 guide). Libraries were quantified by fluorometry, immobilised and processed onto a flow cell with a cBot followed by sequencing using TruSeq v3 chemistry on HiSeq2500 (all components by Illumina (San Diego, CA, USA).

### 2.3. Dual Luciferase Assays

Dual luciferase assays employing 3′UTR reporters in pMIR-RNLTK (a dual firefly and renilla luciferase vector) were carried out in HEK293T cells as described [39]. Alternatively, pGL3 (firefly luciferase) targeted reporters along with phRL (renilla luciferase) (Addgene, Cambridge, MA, USA), transfected in a ratio of 100:1, were conducted in BL2 and BL2/B95-8 cells. Typically 10^5^ HEK293T cells were seeded in 24-well format and transfected using PolyFect (Qiagen, Hilden, Germany) with 0.2 µg/well reporter vector and a total of 0.8 µg effector plasmid(s). Single effector plasmids were used at 0.4 µg/well supplemented with 0.4 µg/well empty pSG5. miRNA expression vectors plus EBER plasmids were transfected at 0.4 µg/well each. For BL cells, 10^7^ cells were transfected by electroporation at 250V, 960F using a Bio-Rad (Munich, Germany) Gene Pulser in 250 µL serum free medium with 10 µg each of reporter and effector plasmids, cultured in multiwell plates and assayed at 48 h. The ratio of firefly (reporter)/renilla (control) luciferase for each sample was determined (%RLU) and assays conducted in triplicate. Statistical significance was tested using Student’s *t*-test.

### 2.4. Plasmids

EBER1/2 was cloned into pSG5 by PCR amplification using oligonucleotide primers listed in Table S1. The ebv-miR-BART16 expression plasmid and the EBER1 expression plasmid p661 (or pLEXIII) have been previously described [36,38]. A derivative of this, including EBER1 promoter sequences from -323 to the immediate EBER1 3’end at nt167 was cloned into a CMV-promoter deleted derivative of pcDNA3.1 (termed here p734). Using p734 as a template, the TTTT sequence at EBER1nt 23 was mutated to TTCC (EBER1:UUCC/p747), also CCTT (EBER1:CCUU/p748) (Table S1). It was subsequently determined that loss of EBER1 upstream and/or downstream sequences (p734 compared to p661) reduced expression levels, therefore the latter 3 plasmids were extended by PCR cloning a further 25 nt upstream (to-348) and 25 nt downstream of the EBER1 stop into pSC-B (Stratagene, now Agilent Technologies, Santa Clara, CA, USA). These extended plasmids are referred to here as p734e, p747e and p748e. Note, in these plasmids, EBER1 expression is driven by the viral pol III EBER1 regulatory sequences. The plasmid pSG70-VA I+II expressing the adenovirus 5 VA transcripts I and II was a generous gift of Stephan Kochanek, Department of Gene Therapy, Ulm University, Ulm, Germany.

### 2.5. Primer Extension

RNA samples were eluted from selected gel fragments in 300 mM NaCl, 10 mM Tris-pH8 at 4 °C overnight. Samples were ethanol precipitated, resuspended in H_2_O and heated briefly to 65 °C. Primer extension was performed using 10 µg eluted RNA and 1 µM radiolabeled 18 mer primer complementary to EBER1 nt 6 to 23, in the presence of 10 mM DTT, 100 µM dNTPs and 50 units Superscript II reverse transcriptase at 42 °C for 1 hr. Products were separated by 15% SDS-PAGE containing 7M urea.

### 2.6. RNA Isolation from Cell Lines/Tissues and Northern Blotting

RNA was extracted from cell line pellets and human tumor biopsy samples using TRIreagent (Sigma-Aldrich, Munich, Germany) or TRIzol (Life Technologies). Samples were DNase treated, further phenol-chloroform extracted and ethanol precipitated. Samples (typically 3 µg) were mixed (1:1) with Ambion gel loading solution and separated by 15% dPAGE in MOPS buffer and 7M urea. Ambion “decade” ssRNA size markers were used (Thermo Fisher Scientific, Grand Island, NY, USA). Gels were ethidium bromide stained, blotted (by semidry electroblotting) and the RNA cross-linked to the membranes using EDC (1-ethyl-3-dimethylaminopropyl carbodimide) at 60 °C for 90 min as described [45]. Blots were prehybridized in 2x SSC, 7% SDS, 200 µg/mL ssDNA at 50 °C. RNA complementary oligomer probes were radiolabeled using the mirVana kit (Life technologies), or full length EBER1 using T7 transcribed from EBER1 template with ^32^P-UTP. Hybridization was at 50 °C overnight. Blots were washed in 0.1 × SSC, 0.1% SDS at 50 °C and visualized using a phosphoimager GE Healthcare Life Sciences, Freiburg, Germany).

### 2.7. La Immunoprecipitation and Detection of Associated RNA

The La-specific serum was generated by rabbit immunization with human recombinant La protein (Eurogentech, Seraing, Belgium). Protein-A-sepharose beads (50 µL, GE Healthcare) were coupled to antibody using 20 µL of La-specific serum (or 20 µL of pre-immune serum as control), overnight in 1 mL PBS. Extracts were prepared from 7 × 10^7^ Raji cells in 3 mL lysis buffer (25 mM Tris-HCl, pH7.4, 150 mM KCl, 2 mM EDTA, 1 mM NaF, 0.5% NP-40, 1 mM DTT, 0.5 mM AEBSF), for 20 min on ice. The lysate (1.4 mL following centrifugation at 17,000 × g, 4 °C, 30 min) was incubated with the coupled beads for 3 h, 4 °C. The beads were collected by centrifugation and washed five times (50mM Tris-HCl, pH 7.4, 300 mM KCl, 1 mM MgCl_2_, 0.5% NP-40). Following proteinase K digestion, the co-immunoprecipitated RNAs were extracted using phenol/chloroform/isoamylalcohol. Total RNA from a 5% aliquot of the lysate volume used for the immunoprecipitations was extracted using TRIzol (Life Technologies) and one-tenth was used for Northern blot analysis. Samples were separated by 12% urea PAGE (National Diagnostics, Atlanta, GA, USA). Synthetic, 5′-end labeled ribo-oligonucleotides served as size markers. The RNA was blotted onto Hybond-N membrane (GE Healthcare) by semidry electroblotting (30 min, 20 V) and cross-linked using EDC for 1 h at 50 °C. DNA oligonucleotides complementary either to EBER1_23_ or to the 3’ end of EBER2 (Table S1) were end labeled by T4 PNK and gamma-^32^P-ATP. Hybridization reactions were performed overnight at 50°C in hybridization buffer (5X SSC, 7% SDS, 20mM Na_2_HPO_4_, 1X Denhardt’s solution). Blots were washed twice in 5 × SSC, 1% SDS and once with 1 × SSC, 1% SDS, at 50 °C and visualized using a phosphoimager. For the detection of La in the cytoplasm, AM29, AM58 and Raji cells were fractionated as described earlier [46].

### 2.8. Western Blotting

HEK293T cells were transfected with 2 µg plasmid(s) 24h after seeding 4 × 10^5^ cells in 6-well dishes. Single plasmids were transfected at 1 µg/well supplemented with 1 µg/well empty pSG5. miRNA expression vectors plus EBER plasmids were co-transfected at 1 µg/well each. Cells were harvested 48 h later. Protein extracts (30 µg) were separated by 12.5% PAGE and transferred to Protran™ membranes (Roth, Karlsruhe, Germany). After blocking, membranes were incubated with the following primary antibodies: rabbit anti-human-La serum (see above), and anti-human-tubulin (Cell Signaling Leiden, The Netherlands) and anti-EBV-EBNA1 [47]. After incubation with appropriate secondary antibodies coupled to horseradish peroxidase (Sigma-Aldrich), the membranes were developed using ECL (life Technologies). For La Western blotting, 100 ng recombinant protein and 10 µg Raji cell lysates were used. The anti-La serum was diluted 1:500 and signals were detected with the LI-COR Odyssey Infrared Imaging System (Lincoln, NE, USA).

### 2.9. ELISA

Twenty-four hours after seeding 10^5^ HaCaTs in 24-well dishes the cells were transfected with 1 µg plasmid(s). Single plasmids were transfected at 0.5 µg/well supplemented with 0.5 µg/well empty pSG5. miRNA expression vectors plus EBER plasmids were co-transfected at 0.5 µg/well each. Supernatants were collected 48h after transfection. Il1α secretion was detected using the human IL-1alpha/IL1F1 Duo Kit (R&D Systems, Minneapolis, MN, USA) according to the manufacturer’s protocol, with a multiplate reader Victor X^TM^ (Perkin Elmer, Waltham, MA, USA) at 450 nm and 550 nm. Data were analyzed using WorkOut 2.5 and the 4-Parameter method.

## 3. Results

### 3.1. EBER RNAs Are Processed to Small Fragments

**Figure 1:**
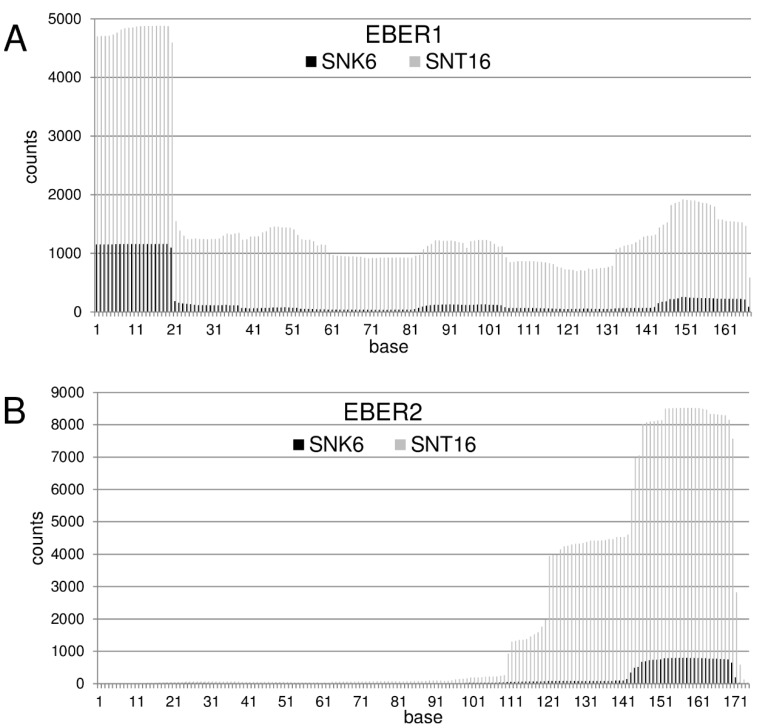
Coverage of EBER reads by sequence analysis of the NK/T-cell lines SNK6/SNT16. After initial quality control, the valid reads of both cDNA-Libraries were mapped to sequences of EBER1 (**A**) and EBER2 (**B**). The counts represent the total number of reads mapped to the particular base positions (black: SNK6, grey: SNT16). Regarding the 5'-end of EBER1 the coverage is dominated by the 20nt sequence from base 1–20, but sequences longer than 20 nt have also been found in much lower abundance. Likewise, the majority of sequences obtained for EBER2 corresponded to nucleotide 143–170, but lower number of reads starting from nucleotide 143, 144 or 146 and ending in 170 or 171 could also be found.

We established the miRNA profile of the EBV-positive NKTL lines SNK6 and SNT16 (Alles *et al.*, submitted). Of the 5,674,444 reads from the SNT16 cell line, 549,121 reads (9.68%) were from EBV-miRNAs. For the SNK6 cell line, we obtained 2,327,119 valid sequences with 160,749 EBV-miRNA reads (6.91%). In the analysis of SNK6 and SNT16 cell small RNA profiles, it was observed that 1.55% and 3.14%, respectively, of the total viral sequence reads were derived from the EBERs (SNK6 cells: 1641 from EBER1 and 886 from EBER2; SNT16 cells: 8,795 from EBER1 and 9001 from EBER2). The 20 nt sequence AGGACCTACGCTGCCCTAGA representing the exact 5′ end of EBER1 was found 915 times in the SNK6 library and 3060 times in the SNT16 library with additional longer fragments extending the 3′ end by 1–4 bases. The most abundant EBER2 fragments were co-terminal with the 3′ end and 25–29 nts in length (nts 143 to 171) (Figure 1).

**Figure 2:**
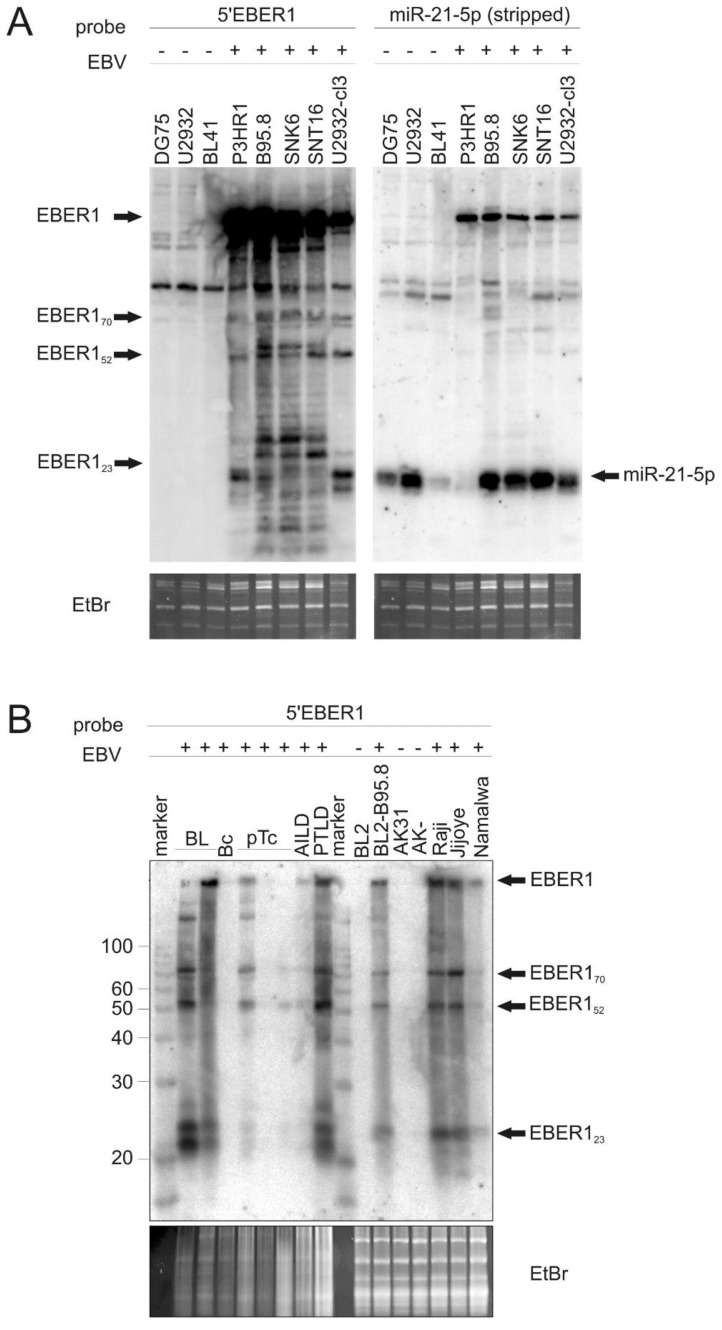
Processing of EBER1 RNA in EBV-infected cell lines and tumors. (**A**) Total RNA of the EBV-negative cell lines DG75, U2932 and BL41 and the EBV-positive cell lines P3HR1, B95-8, SNK6, SNT16 and U2932 cl3 were analyzed by Northern blotting. Left panel: The blot was probed with an oligomer corresponding to nt 1–20 of the 5′ end of EBER1. The position of the processed EBER1-fragments of approximately 70, 52 and 23 nt is indicated. The blot was stripped and re-probed to detect miR-21 to determine the position of a miRNA. Ethidium bromide (EtBr) staining of the gel is shown below to indicate sample loading. (**B**) Identification of processed EBER1 RNA in tumor samples. RNA samples (10 µg) from human EBV-positive tumor biopsies and cell lines were Northern blotted and probed with an oligomer corresponding to the antisense sequence of nt 1–23 of the 5’ end of EBER1. For loading comparison, the ethidium bromide stained gel between 90 and 150 nt (revealing tRNAs, 5SRNA and 5.8SRNA) is shown below. Human primary tumor samples were derived from two Burkitt’s lymphoma (BL), a T-cell rich B-cell lymphoma (Bc), three peripheral T-cell lymphomas (pTc), an AILD sample and a PTLD sample (as indicated). Note: In some cases, particularly the T-cell lymphomas, the EBV may have been resident in tumor infiltrating B-cells and not the neoplastic cells. Three EBV negative cell lines were used: BL2 and two negative derivatives of Akata (AK31 and AK-). Four EBV positive cell lines were examined: BL2/B95-8, Raji, Jijoye and Namalwa. Ss RNA ladder, sizes in nt as indicated.

Furthermore, in an independent study, RNAs from tissues taken from several lines of transgenic mice expressing EBER1 were examined by Northern blotting [36]. These revealed small EBER1 related species detected with an EBER1 5′ probe (data not shown). In order to determine if such small EBER-related products are present in human cell lines and tissues, Northern blot analysis of the SNK6 and SNT16 cells was conducted using a probe corresponding to nucleotides 1–20 of EBER1, here denoted “5′ EBER1”. Simultaneously the EBV-negative cell lines DG75, U2932 and the EBV-infected cell lines P3HR1, B95-8, and the EBV-converted cell line U2932-cl3 [42] were examined (Figure 2A). The blot was stripped and then re-hybridized with a probe for miR-21 (Figure 2A, right panel).

In addition to full-length EBER1, small EBER1 species were present in the EBV-positive cells while miR-21 was detected in all cells. The EBER1 fragments detected were of approximately 23 to 25 nt (migrating slightly above the position of mature miR-21) in P3HR1, B95-8 and U2932-cl3, with slightly larger fragments evident in B95-8, SNK6 and SNT16, as well as larger processed fragments of about 52 and 70 nt in all of the EBV positive cell lines. Subsequently, these RNAs are referred to in this text as EBER1_23_, EBER1_52_ and EBER1_70_. Similar attempts to detect fragments derived from the 3′-end of EBER2 only revealed very faint signals and none were detected using a 5′ EBER2 probe (data not shown).

Using RNAs prepared by a different protocol and separately prepared probes, examination of EBER1 transcripts in the EBV positive BL cell lines Raji, Jijoye and Namalwa, as well as the B95-8 infected BL2 cell line (BL2/B95-8), also consistently revealed EBER1_23_, EBER1_52_ and EBER1_70_ along with full length EBER1, which hybridized to the 5′ EBER1 probe (Figure 2B). Likewise, examination of EBV-positive (of varying positivity) human tumor biopsy samples from multiple tumor types (two Burkitt’s lymphoma (“BL”), one T-cell-rich B-cell lymphoma (“Bc”), three peripheral T-cell lymphomas (pTc), one angioimmunoblastic T-cell lymphoma (“AILD”), one PTLD) revealed a similar array of small EBER1 fragments (note: in some cases, particularly the T-cell lymphomas, the EBV-derived signals may have been resident in tumor infiltrating B-cells and not in the neoplastic cells). These small EBER1 products were detected in all samples in which full length EBER1 could also be detected (the level of EBER1 detected correlates well with the EBV load for the biopsy sample assessed by EBV BamW Taqman analysis; data not shown).

To assess if the observed small EBER1 fragments were not a result of degradation during the preparation of the RNAs, the adenoviral VA I and II transcripts were examined in parallel. The EBERs can functionally replace the VA transcripts in the lytic replication of adenovirus [24] and it was shown previously that the VA transcripts are processed to two short fragments of about 21 and 27 nucleotides from both the 5′ and the 3′ end of VA with RNAi potential [48]. HEK293T cells were transfected in parallel with the EBER1-expression plasmid and a VA I+II expression plasmid and the RNAs analysed the RNAs by Northern blotting (Figure S1). The full- length and the small EBER fragments derived from the 5′ end of EBER1 as well as both the full length and the small 5′ and 3′ fragments of VA I are clearly detectable. This data, together with the detection of the small EBER1 fragments following several different RNA preparation protocols, in different hands and laboratories and using numerous different sample origins, suggest that these small EBER1 fragments are not a result of technical preparation artifact.

**Figure 3:**
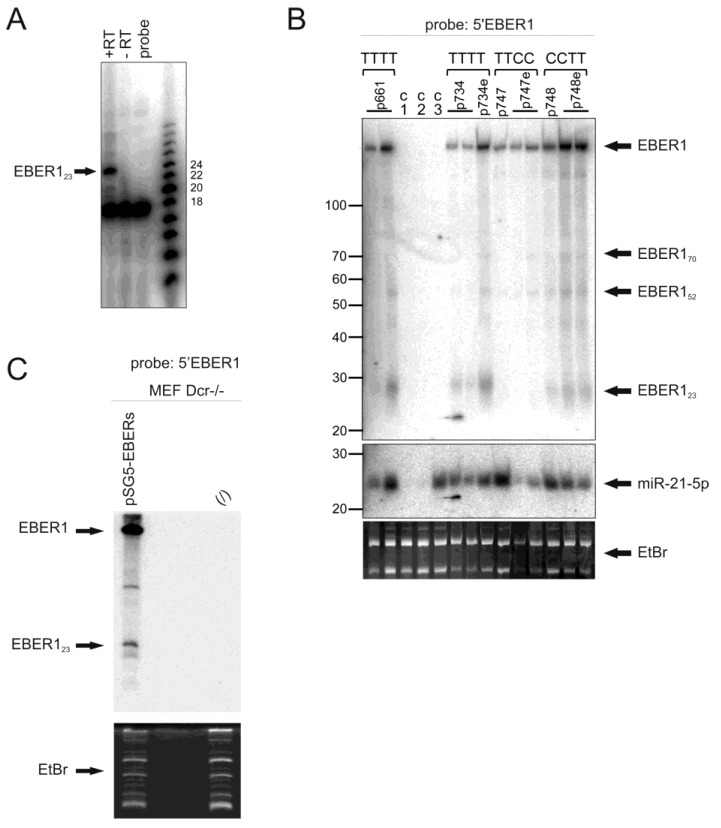
**Processing of EBER1**. (**A**) 5′ end determination of the EBER1_23_ fragment. Primer extension was performed on 10 µg of gel purified EBER1_23_ using an 18mer radiolabeled primer complementary to EBER1 nt 6–23 in the presence or absence of reverse transcriptase (+/-RT). Products were separated on a 15% acrylamide gel, blotted and visualised by phosphoimaging. The probe alone lane shows the 18nt primer. The major 5′extension product is 24 nt (arrow). (**B**) HeLa cells were transfected with various EBER1 expression plasmids (p661, p734, p734e, p747, p747e, p748, p748e, as indicated and detailed in materials), either encoding wild type EBER1 (TTTT), or nts 23 and 24 replaced with C (CCTT), or nts 25 and 26 replaced with C (TTCC). Cells were co-transfected with an expression vector for mutant miR-21. Controls: C1=Hela cells not transfected, C2=transfected with empty vector, C3=transfected with mutant miR-21 alone. RNA was isolated after 24 h, 4 µg samples were separated by dPAGE, blotted and probed with a 5′ EBER1 antisense probe (top panel), then stripped and re-probed with an antisense mutant miR-21 probe (middle panel). For loading comparison, the ethidium bromide stained gel with 5SRNA and 5.8SRNA is shown below. Note, miR-21 is migrating slightly faster than EBER1_23_, relative to the 20 nt and 30 nt markers. Full length EBER1 and the EBER1 fragments are indicated. (**C**) Dicer deficient MEFs were transfected with an EBER1/2 expression vector. Total RNA extracted from transfected and un-transfected cells was subjected to Northern blot analysis and probed with an oligonucleotide complementary to EBER1_23_. Equal loading of the samples is evidenced by ethidium bromide staining of the gel before blotting.

To investigate these EBER1 related RNAs, probes spanning the rest of the EBER1 sequence excluding the 5′ region were used to examine the Raji cell line. Full length EBER1 was detected, but not EBER1_23_ (not shown), suggesting that this RNA originates from the 5′ end of EBER1. Further, an RNA probe specific to sequences located upstream of the known EBER1 5′ end did not hybridise to any of the small EBER1 products (or full length), indicating that the EBER1 fragments do not initiate upstream of the known EBER1 start site. To map these fragments further, 5′ primer extension was conducted with a radiolabeled antisense 18mer oligonucleotide complimentary to nucleotides 6-23 of EBER1. The major extension product of the small EBER1 RNAs was 24 nucleotides long (Figure 3A), indicative of the 5′ end mapping to the published EBER1 start site. The size of EBER1_23_ is similar to miRNAs, however, we were able to detect EBER1_23_ in Dicer-deficient cells [41] transfected with an EBER1/2 expression plasmid (Figure 3C). This indicates that the biogenesis of EBER1_23_ is not mediated by Dicer (as are miRNAs).

The secondary structure of EBER1 is predicted to include double stranded regions with several hairpin loops. The 5′ end closest loop spans nts 20–27 and includes a stretch of four uracil nucleotides from nts 23–26. In order to explore if this stretch acts as a premature termination of pol III transcription signal, site directed mutagenesis was used to change either the first two, or second two thymines to cytosines. These plasmids, along with wild type controls were transfected into HeLa cells (with multiple replicates) and the EBER1 products examined by Northern blotting (Figure 3B and Figure S2). 

The cells were co-transfected with a miR-21 expression vector and the 21nt product migrates slightly faster than EBER1_23_. All three small EBER1 RNAs, EBER1_23_, EBER1_52_ and EBER1_70_, noted in the B-cell lines and tumors were observed, as well as full length EBER1 using the mutant construct EBER1:CCTT, to similar levels as the wild type EBER1:TTTT construct. This suggests that the run of four Ts at this site (altered in the mutant) does not bring about transcriptional termination to generate EBER1_23_. However, expression from the mutant construct EBER1:TTCC revealed EBER1_52_ and EBER1_70_ at similar levels to wild type, while no EBER1_23_ could be detected. It is therefore possible that the small RNAs are products of nuclease processing at the EBER1 structural loops. Mutation of T25 and T26 to cytosine nucleotides in EBER1:TTCC might permit base pairing with the two guanine nucleotides at nt 21 and 22 of the first loop, thus minimising the loop to just 2 bases. This restricted loop in the mutant might limit nuclease activity and explain why no EBER1_23_ is produced. By contrast, C23 and C24 in the EBER1:CCTT mutant would not be able to pair effectively with G21 and G22 due to torsional constraints of the molecule, leaving the loop intact. Larger EBER1-related RNAs, but smaller than full length, could be detected in some samples (Figure 1 and Figure 2), which may reflect nucleolytic products of other loops. Despite this, small EBER1 related RNA fragments were not detected with 3′ probes, suggesting that only the 5′ co-terminal fragments are retained.

### 3.2. EBER1/2 Affects Cellular and Viral miRNA-Guided Repression of Specific mRNAs

**Figure 4:**
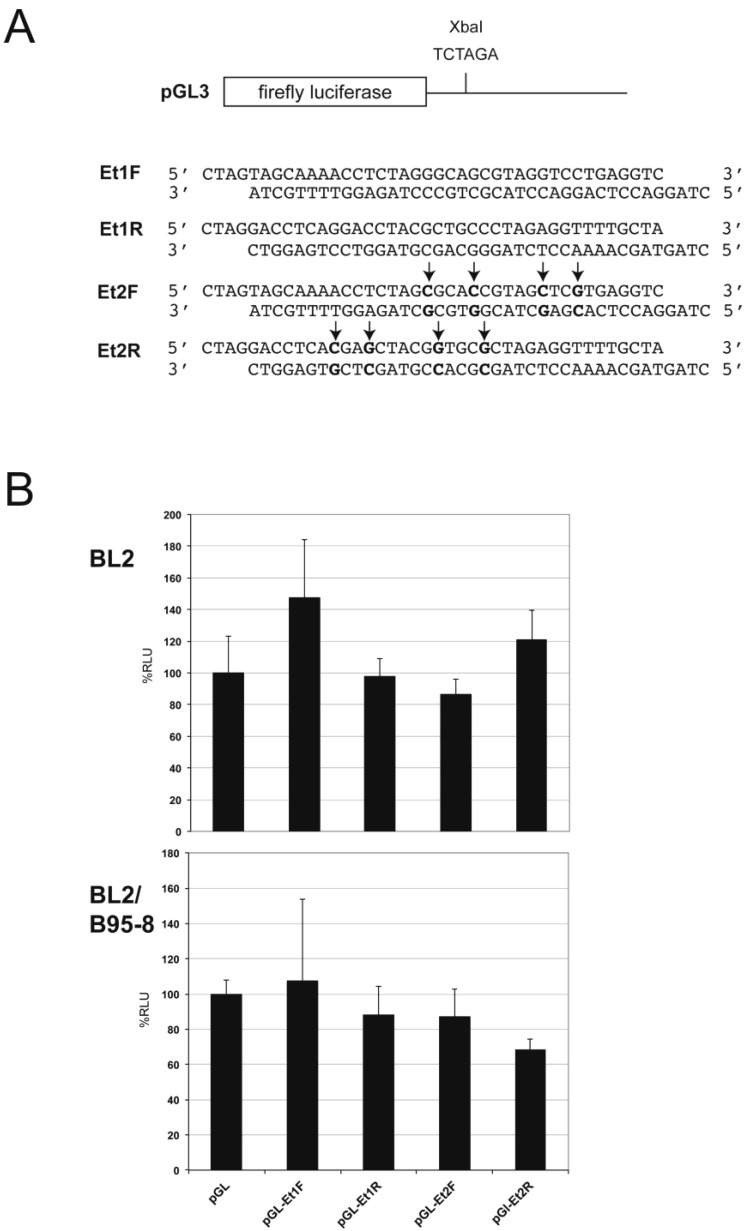
EBER1 and derivatives do not have direct miRNA-like activity. (**A**) Schematic representation of the luciferase EBER1 3′UTR reporter construct generated in the vector pGL3. Sequences shown were inserted at the XbaI site. The Et1F sequence, when transcribed, would be complementary to the 5′end of EBER1 and represents a potential target for EBER1_23_. The reverse (Et1R) has some complementarity to the 3′ end of EBER1. Et2 sequences are the equivalent, but include four-point changes (indicated with arrows). (**B**) EBER1 co-transfection (vector p661) with the luciferase reporter constructs shows no significant difference in relative luciferase activity (%RLU) expressed in either the EBV negative BL cell line BL2 or the EBV positive counterpart (BL2/B95-8). Data shown are from three independent experiments and error bars show standard deviation.

The autoantigen La has been shown to co-activate RNAi by directly binding to Ago2 [49]. In addition, La and La-related proteins are frequently found in RNP isolations including Ago-associated mRNPs [41,50]. Intriguingly, EBER1_23_ is of similar size to miRNAs. In order to explore if EBER1 or its derivatives act in a similar manner to miRNAs, a dual luciferase reporter assay was conducted with firefly luciferase constructs containing an EBER1 targeting sequence (Et1: −5 to +30 of EBER1) in both orientations as well as a four-point mutation version of this sequence (Et2) (Figure 4A). These were assayed with EBER1 co-expression in BL2 (EBV negative) and the virus infected counterpart BL2/B95-8 cells. No significant difference in relative luciferase activity was observed between the targeting constructs and parental vector in either cell line (Figure 4B), indicating that EBER1 (and derivatives) does not act like a miRNA, at least in these cells.

**Figure 5:**
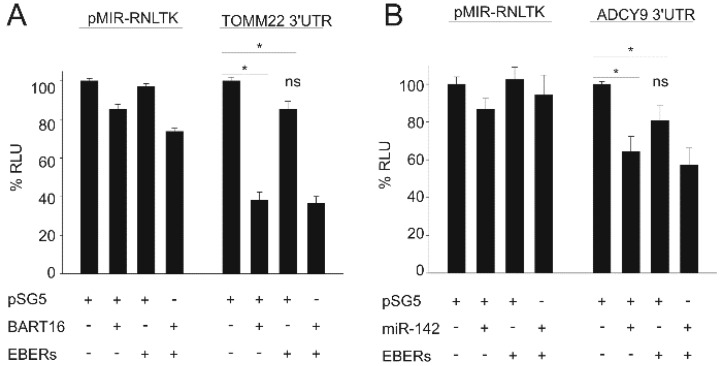

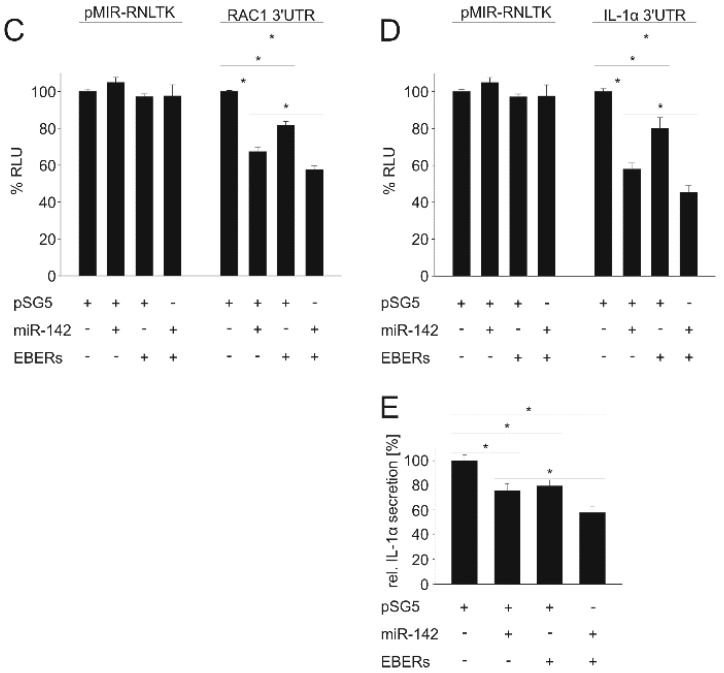
Specific down-regulation of miRNA targets with EBER1/2. (**A**,**B**) EBER1/2 co-expression has no additional influence on the down-regulation of TOMM22 by EBV-miR-BART16 or ADCY9 by miR-142-3p. (**A**) The pMIR dual-luciferase vector pMIR-RNLTK with or without the 3′UTR of the TOMM22 gene was co-transfected with the pSG5-ebv-miR-BART16, the pSG5-EBER1/2 expression vector or the combination of the two as indicated, into HEK293 cells. The left panel shows the effect on the empty reporter vector, the right panel the effect on the pMIR-TOMM22-3′UTR vector (**B**) The pMIR dual-luciferase vector pMIR-RNLTK with or without the 3′UTR of the ADCY9 mRNA was co-transfected with the pSG5-ebv-miR-BART16, the pSG5-EBER1/2 expression vector or the combination of the two as indicated. The left panel shows the effect on the empty reporter vector, the right panel the effect on the pMIR-ADCY9-3′UTR. (**C**,**D**). EBER1/2 reinforce the repression of the RAC1 and IL1α 3′UTR by miR-142-3p. The indicated expression vectors (pSG5 = empty vector) were co-transfected with either the unmodified luciferase reporter vector (pMIR-RNLTK) or incorporating the 3′UTR of IL1α or RAC1 into HEK293 cells. Significant difference in relative luciferase activity (%RLU) (*p* < 0.05) is indicated (*). The graphs represent the results from at least three independent experiments in duplicate. Error bars show SE. (**E**) Influence of miR-142 and EBERs on IL1α protein secretion. The levels of IL1α in the supernatant of HaCaT cells as measured by ELISA, (co-)transfected with the indicated expression vectors is shown. The graph represents the results from at least three independent experiments in duplicate. Significant difference (*p* < 0.05) is indicated (*).

We previously demonstrated that TOMM22 is a target for BART16 [38]. We therefore tested the TOMM22 3′ UTR reporter as described above in various combinations with the BART16 and EBER1/2 expression plasmids. BART16 down-regulation of the TOMM22 3′ UTR was confirmed, however co-expression of the EBERs showed no significant additional effect (Figure 5A). The EBERs were then assayed in a dual luciferase assay in conjunction with miR-142 using the ADCY9, RAC1 and IL1α 3′ UTRs. These were chosen as they are known targets for miR-142-3p [51]. Secondly, we wanted to know if a possible co-regulation by EBER was observed and if this effect was exerted on all targets of a miRNA or on specific targets only. As expected, miR-142 overexpression down-regulated the ADCY9, the RAC1 and the IL1α 3′UTR (Figure 5B–D, respectively). The EBERs had no additional effect upon luciferase activity expressed from the ADCY9 3′-UTR vector (Figure 5B). However, co-expression of the EBERs exerted a significant co-repression on both the RAC1 and IL1α reporters (Figure 5C,D, respectively) (IL1α *p* = 0.02; RAC1 *p* = 0.008). EBER1/2 expression alone also resulted in significant down-regulation of the 3′UTR-reporters, which might suggest these effects are at least partially miR-142-independent or might be mediated by co-operation with other, unknown miRNAs targeting these 3′UTRs. We then analyzed the effect of EBER1/2 co-expression with miR-142-3p on IL1α secretion. Expression of miR-142-3p down-regulated IL1α secretion as previously shown [39] and this effect was significantly enhanced by co-expression of EBER1/2 (Figure 5E). These data suggest that the EBER modulatory activity is selective for specific targets of a given miRNA, in this case miR-142. We also tested a possible effect of the EBERs on the regulation of the IPO7 3′UTR by miR-22, the VEGFA 3′UTR by miR-29b, and ZNF217 by miR-24 [52] but found no additional effect of the EBERs (data not shown). Whether the regulatory mechanism is miRNA-dependent and/or -independent requires further investigation. In summary, our data suggest that the EBERs exert a selective inhibition of gene expression.

### 3.3. The Small EBER1_23_ Fragment Is Bound to the Lupus Antigen (La)

**Figure 6:**
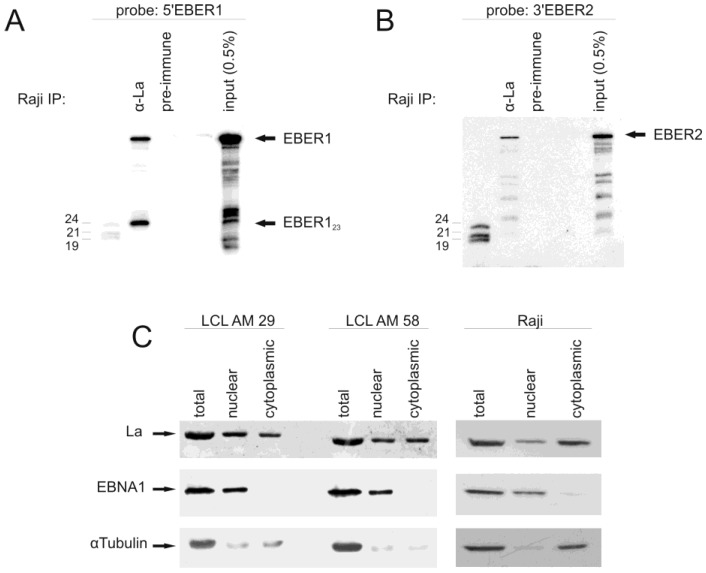
Lupus antigen (La) specifically co-precipitates the small EBER1_23_fragment. (**A**,**B**) Northern blot showing total RNA extracted from Raji cell lysates (input) and RNAs co-purified following immunoprecipitation with anti-La serum (α-La) or with pre-immune serum (pre-immune). The blot was probed sequentially with oligonucleotides complementary to EBER1_23_ (**A**) or to the last 25 nucleotides of the 3′-end of EBER2 (**B**). (**C**) Detection of Lupus antigen in the cytoplasm of EBV-expressing cells. Total cell extract and nuclear and cytoplasmic extracts of lymphoblastoid cell lines transformed by recombinant EBV-wt (“LCL AM 29”) and recombinant EBV deficient for the EBERs (“LCL AM 58”) and the EBV-infected Burkitt’s lymphoma cell line Raji were assayed for the presence of lupus antigen by Western blotting. The presence of EBNA1, which is exclusively nuclear, was assayed to ensure that the cytoplasmic extract did not contain nuclear proteins. Due to the mild lysis conditions, the cytoplasmic alpha-tubulin was detectable in the nuclear fraction, probably resulting from residual non-lysed cells.

It is known that full-length EBERs are not bound to exportin-5 [53]. However, it has been suggested that detection of EBER released from cells is mediated by secretion in exosomes through association with La [22]. Immunoprecipitation of La from Raji cell lysates followed by Northern blot analysis of the co-purified RNAs, revealed precipitated full-length EBER1 as well as EBER1_23_, but not the EBER1_52_ or EBER1_70_ species, indicating selective binding of La to specific EBER1 transcripts (Figure 6). Immunoprecipitation with pre-immune serum did not yield any signal (data not shown). Further, the small EBER1_23_ fragment was enriched compared to full-length EBER1 (Figure 6A, compare signals in input lane to signals in the La immunoprecipitation lane). The RNA precipitated with La was also analyzed for the presence of the 3′ EBER2 fragment identified by sequencing. Binding of full-length EBER2 to La was observed and also to the smaller 3′ EBER2 related fragments, but no relative enrichment of any of these fragments was detected (Figure 6B).

It has been shown that La can regulate the translation of cytoplasmic mRNA [54,55]. To test for the presence of La in the cytoplasm of EBV-infected cells, we generated cytoplasmic and nuclear fractions of the AM29, AM58 and Raji cell lines. Both nuclear and cytoplasmic extracts of all three cell lines clearly contained La protein (Figure 6C). Due to the mild fractionation conditions, some signal for the cytoplasmic alpha-tubulin is detected in the nuclear fraction. However, care was taken to avoid contamination of the cytoplasmic extract with nuclear components and the strictly nuclear viral protein EBV nuclear antigen 1 (EBNA1) is only detected in the nuclear fraction, supporting the conclusion that La exists in both compartments in the cell.

## 4. Discussion

From miRNA profiling of the cell lines SNK6 and SNT16, it was found that EBV-encoded miRNAs overall represented 31.4% and 14.7% of all miRNAs reads. In exploring the small RNAs transcribed from EBV, a relatively large number of fragments derived from the 5′ end of EBER1 were detected and at much lower number, from the 3′ end of EBER2. Northern blot analysis revealed 5′ co-terminal processed species of EBER1 of approximately 23, 52 and 70 nt length in various EBV-infected cell lines, in primary human tumor samples and transgenic mouse tissues. These smaller EBER1 derivatives were consistently detected irrespective of RNA extraction protocol or level of EBER1 expression (very high in some cell lines and low in some tissues). Thus, the derivatives are not generated by the process of cell culture, nor result exclusively from high expression levels. The variety of extraction protocols used (and in several different labs) and sample origins, as well as the lack of detection of 5′ EBER2 derivatives, argues against these fragments resulting from protocol artifact. In addition, the same extraction protocol yielded the small RNA fragments of about 21 and 27 nt already described for the adenoviral VA I transcript which was previously shown to be processed to small fragments with RNAi potential [48]. Primer extension and Northern blot analyses confirmed that EBER1_23_ starts at the reported 5′ end of EBER1. The predicted EBER1 structure comprises several double stranded regions (hairpins) with single stranded loops which span nts 20–27, 50–53, 69–75, 95–101, 116–123, and 139–143. It is possible that EBER1 is sensitive to nucleolytic cleavage within the loops *in vivo*, which is consistent with the size of the fragments detected here and would also explain the slight variability in size observed (if cleavage occurs at different sites within a loop). Site directed mutagenesis studies do not support that the run of four U′s in the first loop direct premature termination of the pol III transcript. However, possible minimization of the loop size (to two nucleotides) does inhibit production of EBER1_23_, which supports the idea that the loops represent nuclease sensitive sites. Despite this, small EBER1 related RNA fragments were not detected with 3′probes, suggesting that only the 5′ co-terminal fragments are retained.

Full length EBER1 and EBER1_23_ were found to be associated with La, however, the other EBER1 small fragments were not bound. This is entirely consistent with the known specificity of La binding to UUU-OH 3′ ends of small RNAs [56]. Like full length EBER1, EBER1_23_ (terminating between nts 23 to 26) would end with a run of uracil nucleotides, while the larger fragments would not. It would also explain the relative greater abundance of EBER1_23_ compared to the other fragments, since La stabilizes bound RNAs and protects them from degradation [57]. The EBER2 fragments derived from the 3′ end of EBER2 were also bound by La, albeit at a low level. Co-terminal with full length EBER2, these would all end in a run of U nucleotides, which would explain La binding and that none showed preferential binding.

Since EBER1_23_ resembles a *bona fide* pol III transcript (with a run of uracils at the 3′ end) and is of similar length to miRNAs, the complement was tested as a potential miRNA target. No direct miRNA activity was observed with EBER1 expression. However, as EBER1_23_ binds to La (along with full length), this raises the possibility that it may be exported in exosomes, as postulated for full length EBER1 [22] and may be a functional RNA species.

While direct miRNA activity was not observed for EBER1 with a 5′EBER1 target sequence, expression of EBER1 and EBER2 together was found to co-operate with miR-142-3p in the down-regulation of the IL1α 3′UTR as well as the RAC1 3′UTR. In addition, the secretion of IL1A protein from transfected cells was also reduced by co-expression of the EBERs supporting the observations based on reporter assays. However, no co-operative down-regulation was observed by the EBERs with the miR-142-3p target ADCY9 or the BART16 target TOMM22 indicating that the EBER regulatory action is neither indiscriminate, nor is it solely directed by a specific miRNA. The luciferase assays used to measure the relative activity of firefly-target/renilla luciferase, thus excluding effects of transfection efficiencies or promoter competition. Additionally, in multiple vector assays, empty vector controls were included (to the same multiplicity of vectors) and in several assays EBER expression was driven by pol III regulatory sequences, unlike the pol II promoters of the target and miRNA vectors, supporting the interpretation that the EBER transcripts selectively modulate gene expression.

Our data are in line with a recent report which shows that the expression of the EBERs in the B-cell line BJAB results in the induction of nine and the down-regulation of 11 proteins [37]. While the up-regulated proteins could be assigned to oncogenic pathways, the down-regulated genes could not be assigned to an overall consensus of biological activity. Wu and colleagues (2007) reported that EBER2, but not EBER1, plays a critical role in transformation by EBV [58], while other reports indicate that EBER1 may contribute to transformation. In our studies, expression of EBER1 and EBER2 together and EBER1 alone show selective modulation of particular targeting vectors, possibly acting in conjunction with the regulating miRNA. It remains unclear whether binding of La to the full length EBERs or EBER products contributes to tumorigenesis or indeed the expression modulation observed here. It has been reported that La associates with Ago2 and helps to release target RNAs after sequence-specific cleavage in RNAi allowing for turnover of RISC [49]. However, Ago proteins also associate with partially complementary sequences and a site-directed, RNAi-like cleavage does not occur. Instead, in the canonical miRNA pathway translation is blocked and mRNA decay induced. It is possible that La might influence these mechanisms as well. This might be supported by the fact that La has been found associated with mRNAs/mRNPs in different species [41,59]. Additionally, La might influence the selective export of miRNA isoforms [13]. However, it is possible that La associates with mRNAs independently of the miRNA machinery and affects gene expression. Such an activity could explain the additive effects of miRNA repression and EBER overexpression, which is observed in our experiments. Furthermore, such a model would also help to explain why not all miRNA targets respond to EBER overexpression. Why La associates with the EBERs (full length EBER1 and -2 and the EBER1_23_ derivative) and whether or not this facilitates miRNA dependent or independent gene expression modulation and/or export, remains to be examined in future experimental approaches.

## Supplementary Files

Supplementary File 1
