# Supplementary material for: Epstein-Barr Virus EBER Transcripts Affect miRNA-Mediated Regulation of Specific Targets and Are Processed to Small RNA Species
Source: Noncoding RNA. 2015 Sep 30;1(3):170–91. doi: 10.3390/ncrna1030170 (PMC5932547; doi:10.3390/ncrna1030170)
Supplement: Supplementary File 1 [file ncrna-01-00170-s001.pdf]

Non-Coding RNA **2015**, *1*, 170-191; doi:10.3390/ncrna1030170

Supplementary Materials

Epstein-Barr Virus EBER Transcripts Affect miRNA-Mediated Regulation of Specific Targets and Are Processed to Small RNA Species

Julia Alles, Daniele Hasler, Syed Mohammad Ali Kazmi, Mathias Tesson, Andrew Hamilton, Linda Schlegel, Stefanie Marx, Norbert Eichner, Richard Reinhardt, Gunter Meister, Joanna B. Wilson and Friedrich A. Grässer

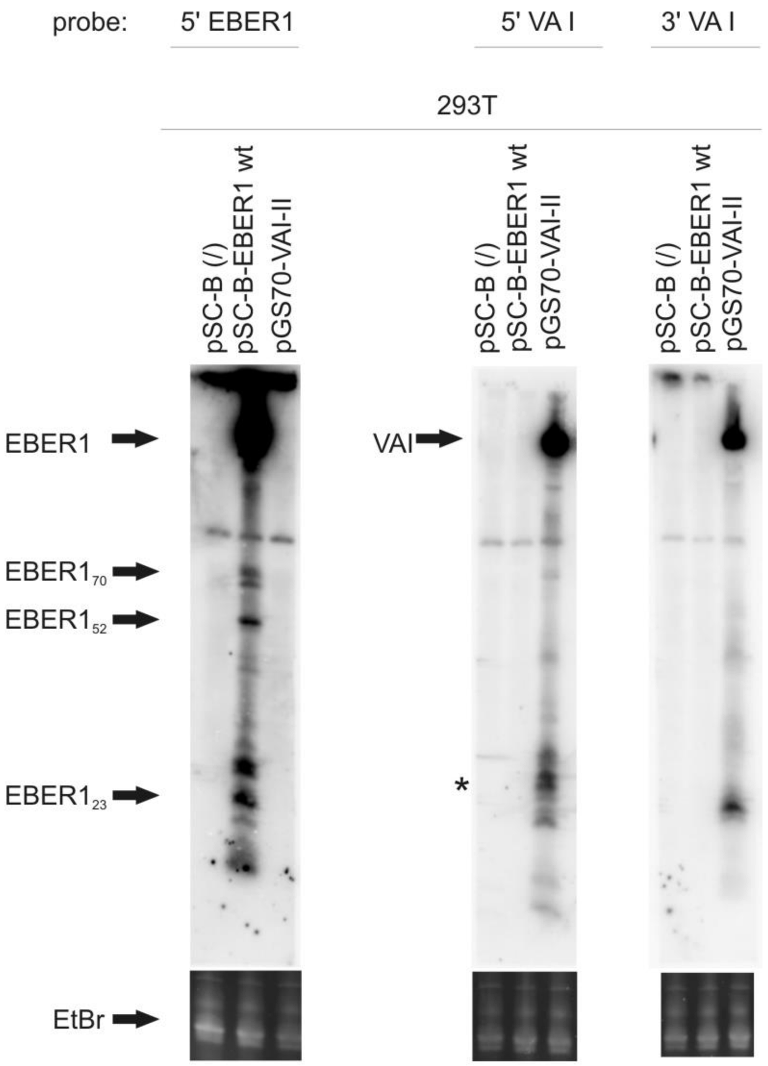

Figure S1. Comparison of the EBER1 and adenoviral VAI small RNA-fragments. HEK293T cells were transfected with either an EBER1 or VA expression vector (pGS70-VA I+II). Transfection of the empty pSC-B vector served as a control. Total RNA was extracted and analyzed by Northern blotting using probes for 5′EBER1, 5′VAI or 3′VAI, as indicated (Table S1). Equal loading of the RNA samples is evidenced by ethidium bromide staining of the gel before blotting (panels below).

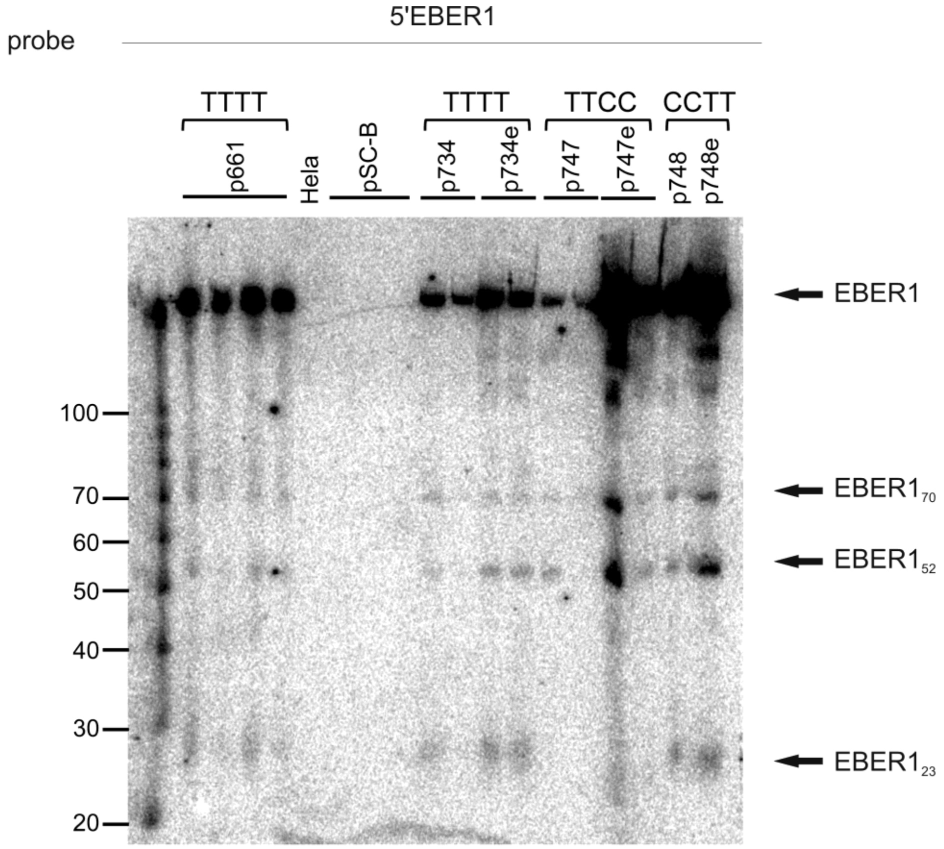

Figure S2. Processing of EBER1 fragments. HeLa cells were transfected with various EBER1 expression plasmids (p661, p734, p734e, p747, p747e, p748, or p748e, as indicated and detailed in Materials), either expressing wild type EBER1 (TTTT), or nts 23 and 24 replaced with C (CCTT), or nts 25 and 26 replaced with C (TTCC). Controls: Hela cells not transfected, or transfected with empty vector (pSC-B). RNA was isolated after 24 h, 4 µg samples were separated by dPAGE and blotted. The blot was probed with a 5′ EBER1 antisense probe. Full length EBER1 (arrow) and the EBER1 fragments are indicated.

Table S1. Sequences of oligonucleotides and probes used in this study

oligo name	sequence
5' EcoRI-EBERs	5'-GGAATTCCCATAGGCAACCCCCGCCTAC-3'
3' BamHI-EBERs	5'-CGGGATCCGGACGGGGTTTCCTGGACACC-3'

sequences of northern blot probes	
**probe name**	**sequence**
5' EBER 1	5'-AGGACCTACGCTGCCCTAGACCTGTCTC-3'
3' EBER 2	5'-AGAAGGGTATTCGGCTTGTCCGCTGTTTCCTGTCTC-3'
5' EBER1 (La-IP)	5'-TCTAGGGCAGCGTAGGTCCT-3'
3' EBER2 (La-IP)	5'-AACAGCGGACAAGCCGAATAC-3'
miR-21	5'-TAGCTTATCAGACTGATGTTGACCTGTCTC-3'
5' VAI	5’-GGGCACTCTTCCGTGGTCTGGCCTGTCTC-3’
3' VAI	5’-AGACAACGGGGGAGTGCTCCTTCCTGTCTC-3’

oligos for extended EBER1 cloning into pSC-B for the expression vectors p743e, p747e and p748e	
**oligo name**	**sequence**
EBER1eF	5'-**GAGCTCCTAGGTCAGACCACGAAAG**GTCAGCCTGCAAGGTGGATGGCG-3'
EBER1eR	5'-**AAATCCTAAAACAAAAGTTTGGATC**AAAACATGCGGACCACCAGCTGG-3'

extended EBV sequence compared to original plasmids (p734, p747 and p748) is marked in bold

EBER1 targeting oligos	
**oligo name**	**sequence**
Et1	5'-ctagGACCTCAGGACCTACGCTGCCCTAGAGGTTTTGCTAgatc-3'
Et2	5'-ctagGACCTCA**C**GA**G**CTACG**G**TGC**G**CTAGAGGTTTTGCTAgatc-3'

DS oligos, where lower case indicates SS overhang

EBER1 site directed mutagenesis oligos (DS)	
**oligo name**	**sequence**
CCTT	5'-GGACCTACGCTGCCCTAGAGG**CCTT**GCTAGGGAGGAGACGTGTGTGG-3'
TTCC	5'-GGACCTACGCTGCCCTAGAGG**TTCC**GCTAGGGAGGAGACGTGTGTGG-3'
